# Addressing the smoking-hypertension paradox in pregnancy: insight from a multiethnic US birth cohort

**DOI:** 10.1097/PN9.0000000000000035

**Published:** 2023-05-24

**Authors:** Henri M. Garrison-Desany, Christine Ladd-Acosta, Xiumei Hong, Guoying Wang, Irina Burd, Zila van der Meer Sanchez, Xiaobin Wang, Pamela J. Surkan

**Affiliations:** 1 Department of Epidemiology, Johns Hopkins Bloomberg School of Public Health, Baltimore, MD, USA; 2 Center on the Early Life Origins of Disease, Department of Population, Family and Reproductive Health, Johns Hopkins University Bloomberg School of Public Health, Baltimore, MD, USA; 3 Integrated Research Center for Fetal Medicine, Department of Gynecology and Obstetrics, Johns Hopkins University School of Medicine, Baltimore, Maryland, USA; 4 Escola Paulista de Medicina, Universidade Federal de São Paulo, São Paulo, São Paulo, Brazil; 5 Department of Pediatrics, Johns Hopkins University School of Medicine, Baltimore, MD, USA; 6 Department of International Health, Johns Hopkins Bloomberg School of Public Health, Baltimore, MD, USA.

**Keywords:** Hypertension, Smoking, Pregnancy complications, Perinatal epidemiology, Cardiovascular risks

## Abstract

**Background:**

Smoking during pregnancy has been associated with reduced risk of a spectrum of hypertensive (HTN) disorders, known as the “smoking-hypertension paradox.”

**Objective:**

We sought to test potential epidemiologic explanations for the smoking-hypertension paradox.

**Methods:**

We analyzed 8510 pregnant people in the Boston Birth Cohort, including 4027 non-Hispanic Black and 2428 Hispanic pregnancies. Study participants self-reported tobacco, alcohol, cannabis, opioids, or cocaine use during pregnancy. We used logistic regression to assess effect modification by race/ethnicity, and confounding of concurrent substances on hypertensive disorders or prior pregnancy. We also investigated early gestational age as a collider or competing risk for pre-eclampsia, using cause-specific Cox models and Fine-Gray models, respectively.

**Results:**

We replicated the paradox showing smoking to be protective against hypertensive disorders among Black participants who used other substances as well (aOR: 0.61, 95% CI: 0.41, 0.93), but observed null effects for Hispanic participants (aOR: 1.14, 95% CI: 0.55, 2.36). In our cause-specific Cox regression, the effects of tobacco use were reduced to null effects with pre-eclampsia (aOR: 0.81, 95% CI: 0.63, 1.04) after stratifying for preterm birth. For the Fine-Gray competing risk analysis, the paradoxical associations remained. The smoking paradox was either not observed or reversed after accounting for race/ethnicity, other substance use, and collider-stratification due to preterm birth.

**Conclusions:**

These findings offer new insights into this paradox and underscore the importance of considering multiple sources of bias in assessing the smoking-hypertension association in pregnancy.

## Background

Hypertensive disorders during pregnancy remain a leading cause of maternal mortality during delivery.^[1]^ Hypertensive disorders are defined as (a) prior chronic hypertension that continues into pregnancy, (b) pre-eclampsia/eclampsia, (c) pre-eclampsia superimposed on chronic hypertension, or (d) gestational hypertension.^[2]^ Long-term post-delivery effects lead to maternal morbidity, including coronary artery disease, congestive heart failure, and mortality.^[3]^ Similarly, children born to people with a hypertensive disorder in pregnancy are more likely to experience vascular complications and to be hospitalized by age one.^[4]^ Despite known sequelae and intervention efforts, the overall rate of hypertensive disorders in pregnancy has remained high, and hypertension is the second most common cause of maternal mortality in the world.^[5]^ Thus, understanding risk factors for hypertensive disorders remains a pressing issue.

Smoking is an established risk factor for hypertension^[6]^; however, there is a reported “paradoxical relationship” whereby smoking during pregnancy is related to lower likelihood of pregnancy hypertensive disorders.^[7–10]^ This smoking-hypertension paradox has been widely documented,^[8,9,11–13]^ suggesting it is not driven by statistical chance. But given that it is extremely unlikely that smoking is protective during pregnancy, there are likely other epidemiological phenomena at play.^[14]^ Generally, there are three epidemiological explanations why we may see an association between an exposure and outcome, due to either confounding, collider stratification, or due to a causal effect.^[14]^ Notably, selection biases (including left truncation or live birth bias, when an individual has already had an intermediate outcome, such as stillbirth, prior to the study start) can also be conceptualized as collider-stratification bias, whereby two variables have the same common cause that is conditioned upon, and in our case, a variable associated with selection is always conditioned on for entry into the cohort. Additionally, there may be confounding of this relationship potentially due to (a) concurrent substance exposures or (b) parity, since patients who smoked in a prior pregnancy resulting in a hypertensive disorder may be less likely to smoke in subsequent pregnancies. There may also be (c) colliding of gestational age at delivery, or (d) competing risks from other smoking-related outcomes, such as preterm birth (PTB). If smoking tobacco increases the risk of PTB and hypertension, then PTB may be a competing risk since the latter cannot be diagnosed if the child is born early. Alternatively, if both smoking and hypertension increase the risk of PTB, then a collider-stratification bias may be opened when controlling for gestational age in models or conditioning selection on term births. Finally, there may be (e) effect modification from sociodemographic factors such as race/ethnicity, which would essentially serve as a form of collider stratification based on selection bias. This is due to effect modification essentially defining the population selected for (and whether there are differences between the selected populations).

Given the nuance in these cases, conceptually driven hypotheses must be considered and tested. Using these five hypotheses, the overall aim of this study was to gain new insight into reasons for the smoking-pregnancy hypertension paradox in a multiethnic, predominantly low-income birth cohort at increased risk for both hypertensive disorders and substance use (including tobacco smoking).^[15–18]^ The Boston Birth Cohort (BBC) is also enriched for PTB, allowing us to examine competing risks in this population. We examine the impact of these factors on both hypertensive disorders overall (including chronic hypertension), as well as pre-eclampsia to understand the smoking-hypertension paradox during pregnancy and address potential biases that may lead to it in research.

## Methods

### Study sample

We used data from the BBC, a multiethnic birth cohort, which has previously been described.^[19]^ Briefly, beginning in 1998, participants were recruited within 24 and 72 hours after giving birth at Boston Medical Center to live children, a large safety net hospital, and provided written informed consent for themselves and their children were enrolled in the study. They completed a face-to-face interview with data collectors using a standardized study questionnaire at baseline, and follow-up was conducted via passive ascertainment from the maternal and child electronic medical records (EMRs) with International Classification of Diseases (ICD)-9^[20]^ and ICD-10 codes^[21](p10)^ recorded for medical billing. For this secondary analysis, we used data collected at the baseline study visit, including 8510 distinct live births. The study was conducted in accordance with the 1964 Declaration of Helsinki and its recent updates through 2013, and comparable ethical standards. Written informed consent was obtained for all participants in the study.

### Exposure

Self-reported substance use was defined as use of the following in any trimester of pregnancy: (a) tobacco smoking, (b) alcohol consumption, (c) crack or powder cocaine (“cocaine”), (d) heroin, methadone, or non-prescription oxycodone use (“opioids”), and (e) cannabis. While it is important for pregnant people receiving opioid agonist therapy to continue treatment during pregnancy, we hypothesize a similar biological mechanism of action with non-prescription opioids. Also, ICD codes for neonatal abstinence syndrome (NAS) and neonatal opioid withdrawal syndrome (NOWS) from the child’s medical record (ICD-9: 779.5, ICD-10: P96.1) were also coded as opioid exposure during gestation.

We generated a count variable of substances used during pregnancy (regardless of which substances). The scale ranged from no substance use (a score of 0), a score of 1 for using a single substance, to use of all five substances during pregnancy (a score of 5). Notably, this score does not reflect a similar direct mechanism of action, given the differences in the biological effects of these substances. Rather, this variable reflects an indirect measure of the factors that may result in an individual using an increasing number of substances during pregnancy. In a separate model, we examined interactions between any smoking during pregnancy and other substances, defined as: (a) no substance use, (b) only use of smoking tobacco during pregnancy, (c) use of only other substances during pregnancy, (d) smoking and use of at least one other substance during pregnancy.

### Outcome

Hypertensive (HTN) disorders during pregnancy were defined by diagnosis in the medical record using the American College of Obstetrics and Gynecology definition of (a) pre-eclampsia/eclampsia (ICD-9 code 642.4x, 642.5x, 642.7x, ICD-10 code O14.x, O15.x), (b) pre-existing chronic hypertensive disorders (ICD-9 code 642.0x, ICD-10 code O10.x), (c) chronic hypertensive disorders with pre-eclampsia (ICD-9 code 642.7x, ICD-10 code O11.x), and (d) gestational hypertension (ICD-9 code 642.3, ICD-10 code O13.x).^[22]^ We also examined pre-eclampsia specifically, but were not well-powered to examine eclampsia. Gestational hypertension was also investigated (Supplementary Tables, http://links.lww.com/PN9/A22), but is a less severe outcome that may have misclassification.^[23]^ Additionally, hemolysis, elevated liver enzymes, low platelet count (HELLP) syndrome was collinear with pre-eclampsia—only 14 pregnancies had HELLP exclusively—therefore, we did not study this separately.

### Covariates

Covariates included self-reported maternal education (elementary school, some secondary school, graduation from secondary school, some college, and graduation from college); yearly income quartile (including a category “did not know”), maternal age at delivery, pre-pregnancy body mass index (BMI) defined as weight (kilograms) divided by height squared (meters), country of birth (US or foreign-born), parity, child sex, and self-reported stress assessed during the index pregnancy, and general life stress before pregnancy.^[24]^

PTB was defined as birth occurring at gestational age <37 weeks determined from first trimester ultrasound (<20 weeks) or based on the first day of the last menstrual period as recorded in maternal EMRs (if early prenatal ultrasound was not available) with or without medical inducement or other indication. Gestational age at delivery was the time metric in the Cox proportional hazards model.

### Statistical analyses

We generated descriptive statistics across each race/ethnicity. We used χ^2^ tests to examine univariate associations between covariates and race/ethnicity, as well as substance exposures and hypertensive outcomes. Analysis of variance (ANOVA) tests were conducted for continuous variables across racial/ethnic categories. We used logistic regression to generate odds ratios (ORs), 95% confidence intervals (CIs), and *P* values. We estimated associations between substance exposure and HTN, and substance exposure and pre-eclampsia, specifically. Models were adjusted for maternal age, pre-pregnancy BMI, yearly income, education, country of birth, parity, and sex of the child.

### Assessment of Hypotheses

We generated directed acyclic graphs (DAGs) to formalize the three main hypothesized scenarios that we test in this study.

#### Confounding by indication via concurrent substance exposure and prior pregnancies

We hypothesized there may be confounding by indication due to concurrent substance exposure. For instance, exposure to cocaine is known to be associated with tobacco exposure,^[25–27]^ and is a likely risk factor for pre-eclampsia.^[28]^ Therefore, we examined each substance exposure as a potential confounder via regression models.

We also hypothesized that there may be confounding by indication of HTN in a prior pregnancy, and pregnant people who previously had pre-eclampsia or another hypertensive disorder may take precautions to reduce their risk in subsequent pregnancies. Therefore, we assessed the association of interest while restricting our sample to primiparous individuals to assess confounding by this indication. We hypothesized that participants who previously had children may have had pre-eclampsia or gestational hypertension during their prior pregnancy, and, therefore, may reduce substance use in their later pregnancies.

#### Competing risks and collider bias

We hypothesized the possibility of collider stratification with pre-eclampsia due to PTB. We considered it likely that participants who deliver early do not develop pre-eclampsia later in pregnancy. Given studies that either determine eligibility based on term births or control for gestational age in their models,^[29,30]^ this may result in collider stratification bias due to selection or due to an open pathway from this model adjustment with gestational age. Therefore, we calculated the cause-specific Cox proportional hazards ratio for substance use across two outcomes: study participants having spontaneous PTB and study participants having pre-eclampsia. The proportionality assumption was assessed using Schoenfeld residual estimates.^[31]^

We also examined the effect of substance use on hypertensive disorders using the Fine and Gray subdistribution hazards model^[32]^ to account for the competing risk of PTB. We then conducted sensitivity analyses using logistic regression models and a composite outcome for either having hypertensive disorders or SPTB.

#### Effect modification by race/ethnicity

Given prior evidence that the risk of smoking and hypertensive disorders vary due to race/ethnicity,^[33]^ we hypothesized that this may also be the case in our sample. We stratified our models to assess potential effect modification by race/ethnicity. We report the stratified results among White participants in the Supplement, as there was reduced power to determine substance use effects.

### Missingness

Missingness patterns were assessed and multiple imputation by chained equation (MICE) was conducted for variables as appropriate. The yearly income “don’t know” category was considered not missing at random, and so included as one of the imputation categories, rather than reassigning those individuals to other income categories. With MICE, 10 datasets were generated with 50 iterations each.

### Sensitivity analyses

For sensitivity analyses, we further stratified each race/ethnicity group by US-born vs. foreign-born participants based on prior literature suggesting substance use patterns and hypertension risks differ based on immigration status.^[33,34]^ We also stratified by fetal sex. We further conducted E-value analysis to estimate the effect size of confounders necessary to negate our main findings (Supplementary Tables 8 and 9, http://links.lww.com/PN9/A22).

### Replication of hazards models

To replicate our primary finding in an additional cohort, we used the National Vital Statistics System (NVSS) Birth Data available for 2020.^[35]^ The NVSS data has previously been described.^[36]^ A subset (*n* = 24,790) was used for our sample that included study participants having live births and had no missing data for the following variables: gestational hypertension, cigarette use in pregnancy, maternal race/ethnicity, maternal education, maternal age, maternal pre-pregnancy body-mass-index, number of prior live births, and the child’s sex at birth. Given fewer variables are collected through the NVSS compared to the BBC, we focused this brief replication on Cox proportional hazards and Fine-Gray Subdistribution hazards models to examine whether our collider-stratification findings held. Other substance use data during pregnancy beyond cigarette smoking, or additional hypertension data were not available in the NVSS dataset. Therefore, we report these findings for solely tobacco and its associations with gestational hypertension in Supplementary Materials, http://links.lww.com/PN9/A22.

## Results

**Figure 1:**
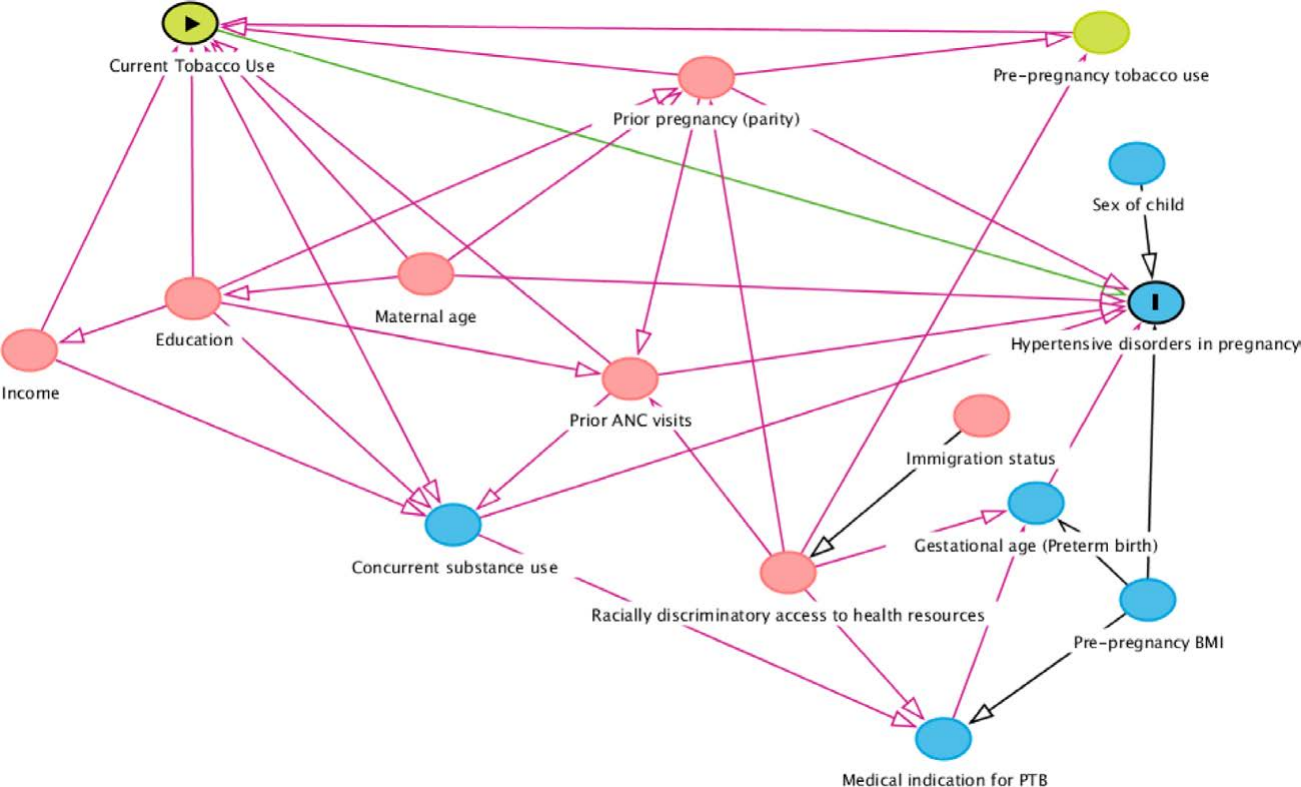
Directed acyclic graph (DAG) of hypothesized relationships between exposure, outcomes, confounders, and covariates.

We created a primary DAG to identify important risk and protective factors in the relationship of interest (Figure 1). In our analytic sample, 8,510 total participants had substance use exposure and outcome data available (Table 1). The plurality of participants identified as Non-Hispanic Black (*n* = 4027, 47.3%), followed by Hispanic (*n* = 2428, 28.6%). In our sample, 12.2% (*n* = 1032) of participants smoked tobacco at some point during pregnancy. However, 15.1% of Black participants (*n* = 604) and 12.8% (*n* = 309) Hispanic participants smoked, while most White mothers (25.1%, *n* = 252) reported tobacco use during pregnancy. Additionally, <5% of Black and Hispanic participants reported using opioids during pregnancy (*n* = 30, 0.7%; *n* = 35, 1.4%, respectively), while opioid use was present in over a quarter of White participants (25.6%, *n* = 258).

**Table 1:** Descriptive characteristics of participants by self-reported race/ethnicity.

	Total sample (*n* = 8510)*	Non-Hispanic Black (*n* = 4027, 47.3%)	Hispanic (*n* = 2428, 28.6%)	Non-Hispanic White (*n* = 1007, 11.8%)	*P* value
Education					<0.001
Elementary school only	546 (6.4%)	85 (2.1%)	406 (16.7%)	16 (1.6%)	
Some secondary school	2186 (25.7%)	882 (21.9%)	900 (37.1%)	180 (17.9%)	
High school graduate	2825 (33.2%)	1472 (36.6%)	664 (27.3%)	362 (35.9%)	
Some college	1659 (19.5%)	216 (23.6%)	308 (12.7%)	216 (21.4%)	
Completed college or higher education	1294 (15.2%)	638 (15.8%)	150 (6.2%)	233 (23.1%)	
Yearly income category					<0.001
<$15,000/year	2687 (31.6%)	1349 (33.5%)	756 (31.1%)	304 (30.2%)	
≥$15,000–<$30,000/year	1632 (19.2%)	852 (21.2%)	440 (18.1%)	136 (13.5%)	
≥$30,000–<$50,000/year	677 (8.0%)	336 (8.3%)	132 (5.4%)	97 (9.6%)	
≥$50,000/year	480 (5.6%)	146 (3.6%)	56 (2.3%)	195 (19.4%)	
Do not know	3034 (35.7%)	1344 (33.4%)	1044 (43.0%)	275 (27.3%)	
Immigration					<0.001
Born in the US	5213 (61.3%)	2244 (55.7%)	1962 (80.8%)	174 (79.5%)	
Born outside the US	3292 (38.7%)	1781 (44.2%)	463 (19.1%)	833 (20.5%)	
Refuse to answer	5 (0.1%)	2 (0.005%)	3 (0.1%)	0	
Maternal age at delivery (Median, IQR)	27.8 (23.0, 32.9)	28.4 (23.1, 33.6)	26.5 (22.2, 31.6)	28.0 (24.0, 32.6)	<0.001
Pre-pregnancy BMI (Median, IQR)	24.7 (21.6, 29.0)	25.7 (22.3, 30.0)	24.5 (21.8, 28.3)	23.5 (20.9, 27.5)	<0.001
Parity					<0.001
Primiparous	4847 (57.0%)	2375 (59.0%)	1438 (59.2%)	493 (49.0%)	
Multiparous	3663 (43.0%)	1652 (41.0%)	990 (40.8%)	514 (51.0%)	
Sex of child					0.74
Male	4370 (50.0%)	2053 (49.7%)	1248 (50.0%)	512 (49.6%)	
Female	4364 (50.0%)	2075 (50.3%)	1246 (50.0%)	520 (50.4%)	
Tobacco					<0.001
Never	6097 (32.4%)	2990 (74.9%)	1968 (81.8%)	325 (32.4%)	
Only before pregnancy	1313 (15.6%)	604 (15.1%)	309 (12.8%)	252 (25.1%)	
Smoked during pregnancy	1032 (12.2%)	399 (10.0%)	130 (5.4%)	427 (42.5%)	
Alcohol					<0.001
None during pregnancy	7747 (91.0%)	3682 (91.4%)	2266 (93.3%)	841 (83.5%)	
Drank during pregnancy	763 (9.0%)	345 (8.6%)	162 (6.7%)	166 (16.5%)	
Cannabis					<0.001
None during pregnancy	8178 (96.1%)	3821 (94.9%)	2391 (98.5%)	951 (94.4%)	
Cannabis during pregnancy	332 (3.9%)	206 (5.1%)	37 (1.5%)	56 (5.6%)	
Opioids					<0.001
None during pregnancy	8170 (96.0%)	3997 (99.3%)	2393 (98.6%)	749 (74.4%)	
Opioids during pregnancy	340 (4.0%)	30 (0.7%)	35 (1.4%)	258 (25.6%)	
Cocaine					<0.001
None during pregnancy	8420 (98.9%)	3991 (99.1%)	2412 (99.3%)	971 (99.8%)	
Cocaine during pregnancy	90 (1.1%)	36 (0.9%)	16 (0.7%)	36 (0.2%)	
Any substance use during pregnancy†	2167 (25.5%)	945 (23.5%)	382 (15.7%)	633 (62.9%)	<0.001
Smoking and additional substances					<0.001
Only smoking during pregnancy	952 (10.9%)	233 (22.6%)	174 (7.0%)	430 (15.2%)	
Any substances except tobacco during pregnancy	568 (6.5%)	113 (10.9%)	132 (5.3%)	211 (7.5%)	
Smoking and at least 1 additional substance during pregnancy	730 (8.4%)	302 (29.3%)	95 (3.8%)	277 (9.8%)	
Illicit substance use during pregnancy‡	656 (7.7%)	248 (6.2%)	72 (3.0%)	290 (28.8%)	<0.001
Hypertension	1446 (17.0%)	815 (20.2%)	325 (13.4%)	138 (13.7%)	<0.001
Pre-eclampsia	832 (9.8%)	459 (11.4%)	216 (8.9%)	68 (6.8%)	<0.001
Spontaneous preterm birth	1522 (17.9%)	736 (18.3%)	401 (16.5%)	213 (21.2%)	0.006
Medically indicated preterm birth	800 (9.2%)	420 (10.2%)	189 (7.6%)	87 (8.4%)	0.004
Gestational age (weeks, median (IQR))	38.9 (36.7, 40.1)	38.6 (36.6, 40.1)	39.0 (37.0, 40.1)	38.7 (36.4, 40.0)	<0.001

*P* value reflects χ^2^ tests for categorical variables and one-way ANOVA tests for continuous variables.

* We report non-Hispanic Black, Hispanic, and non-Hispanic white subgroups, however an additional 1048 individuals reported race/ethnicity as: Asian, Pacific Islander, Cape Verdean, Native American/American Indian, Alaskan Native, multiracial/mixed race, and other categories. These additional groups did not have sufficient sample size as stratified categories.

† Smoking, alcohol, cannabis, opioids, or cocaine.

‡ Cannabis, opioids, or cocaine (regardless of drinking alcohol or smoking tobacco during pregnancy).

We stratified the analysis by non-Hispanic Black (Table 2), Hispanic participants (Table 3), and White participants (Supplementary Table 1, http://links.lww.com/PN9/A22). Adjusting for confounders, Black participants who used opioids had 3.24 increased odds (95% CI: 1.34, 7.84) of hypertensive disorders compared to those who did not use opioids in pregnancy, and similar direction of effect when examining pre-eclampsia, though this overlapped with the null. Tobacco smoking had a negative direction of effect but was not significantly associated with hypertensive disorders (OR: 0.86, 95% CI 0.64, 1.15) or pre-eclampsia (OR: 0.85, 95% CI: 0.61, 1.17) after adjustment for covariates and additional substance use. However, for polysubstance use category, the use of both substances significantly reduced the odds of hypertensive disorders by 39% (OR: 0.61, 95% CI: 0.41, 0.93).

**Table 2:** Substance use during pregnancy and association with hypertensive disorders and pre-eclampsia among Non-Hispanic Black participants (*n* = 4027).

	Hypertensive disorders*		Pre-eclampsia (mild or severe)	
Unadjusted models	Odds ratio (95% CI)	*P* value	Odds ratio (95% CI)	*P* value
Opioids†	4.09 (1.76, 9.49)	0.001	1.98 (0.73, 5.33)	0.17
Cocaine	0.26 (0.07, 0.99)	0.05	0.20 (0.03, 1.52)	0.12
Cannabis	0.80 (0.53, 1.21)	0.29	0.99 (0.61, 1.61)	0.97
Alcohol	1.08 (0.79, 1.47)	0.63	1.44 (1.03, 2.03)	0.03
Tobacco	0.91 (0.71, 1.67)	0.46	0.83 (0.62, 1.11)	0.20
Polysubstance score‡	0.91 (0.79, 1.05)	0.19	0.95 (0.80, 1.13)	0.57
Polysubstance category§				
Neither	REF		REF	
Smoking, no other substances	1.01 (0.77, 1.34)	0.92	0.94 (0.67, 1.31)	0.72
No smoking, other substances	1.17 (0.90, 1.53)	0.24	1.50 (1.11, 2.02)	0.01
Both	0.72 (0.51, 1.02)	0.07	0.78 (0.52, 1.19)	0.26
	Hypertensive disorders*		Pre-eclampsia (mild or severe)	
Adjusted models∥	Odds ratio (95% CI)	*P* value	Odds ratio (95% CI)	*P* value
Opioids	3.24 (1.34, 7.84)	0.001	1.88 (0.69, 5.15)	0.22
Cocaine	0.39 (0.10, 1.56)	0.18	0.25 (0.03, 1.92)	0.18
Cannabis	0.83 (0.52, 1.34)	0.45	1.00 (0.59, 1.69)	0.99
Alcohol	0.96 (0.67, 1.36)	0.80	1.40 (0.96, 2.03)	0.08
Tobacco	0.86 (0.64, 1.15)	0.30	0.85 (0.61, 1.17)	0.32
Polysubstance score‡	0.84 (0.71, 1.00)	0.05	0.90 (0.74, 1.10)	0.31
Polysubstance category§				
Neither	REF		REF	
Smoking, no other substances	0.82 (0.57, 1.17)	0.28	0.82 (0.56, 1.22)	0.33
No smoking, other substances	1.13 (0.82, 1.56)	0.45	1.36 (0.95, 1.95)	0.09
Both	0.61 (0.41, 0.93)	0.02	0.70 (0.44, 1.11)	0.13

Odds ratios demonstrate the associated odds of hypertensive disorders/pre-eclampsia among participants using compared to those not using (reference) a given substance.

* Hypertensive disorders include pre-eclampsia, eclampsia, gestational hypertension, and chronic hypertension.

† Substance use effects were estimated controlling for concurrent substance use, each model included the 4 other substances used during pregnancy.

‡ The polysubstance score was defined as the count of substances used (reference was using 0 substances during pregnancy, ranging to using all 5 substances during pregnancy), controlling for only sociodemographic covariates.

§ Polysubstance use categories were defined as: (1) using no substances during pregnancy (“neither”), (2) only smoking tobacco during pregnancy but no other substances, (3) using opioids, cocaine, or alcohol during pregnancy but no smoking, or (4) smoking and using either opioids, cocaine, or alcohol during pregnancy (“both”).

∥ Adjusted for maternal age, maternal pre-pregnancy BMI, yearly income, educational status, immigration history, parity, and sex of the child. BMI, body mass index; CI, confidence interval.

**Table 3:** Substance use during pregnancy and association with hypertensive disorders and pre-eclampsia among Hispanic participants (*n* = 2428).

	Hypertensive disorders*		Pre-eclampsia (mild or severe)	
Unadjusted models	Odds ratio (95% CI)	*P* value	Odds ratio (95% CI)	*P* value
Opioids†	1.03 (0.25, 4.22)	0.97	0.63 (0.12, 3.17)	0.58
Cocaine	0.41 (0.04, 4.13)	0.45	0.87 (0.10, 8.52)	0.91
Cannabis	1.75 (0.69, 4.42)	0.24	2.28 (0.87, 5.98)	0.09
Alcohol	0.74 (0.44, 1.27)	0.28	0.88 (0.49, 1.60)	0.68
Tobacco	0.82 (0.51, 1.32)	0.40	0.86 (0.51, 1.43)	0.51
Polysubstance score‡	0.87 (0.67, 1.15)	0.33	0.95 (0.71, 1.28)	0.75
Polysubstance category§				
Neither	REF		REF	
Smoking, no other substances	0.65 (0.37, 1.16)	0.15	0.61 (0.32, 1.18)	0.14
No smoking, other substances	0.63 (0.37, 1.09)	0.10	0.67 (0.36, 1.26)	0.21
Both	1.00 (0.55, 1.82)	1.00	1.35 (0.73, 2.52)	0.34
	Hypertensive disorders*		Pre-eclampsia (mild or severe)	
Adjusted models∥	Odds ratio (95% CI)	*P* value	Odds ratio (95% CI)	*P* value
Opioids	1.34 (0.25, 7.21)	0.74	0.60 (0.11, 3.38)	0.56
Cocaine	0.28 (0.02, 3.88)	0.34	0.80 (0.07, 9.14)	0.86
Cannabis	2.66 (0.92, 7.64)	0.07	2.69 (0.91, 7.92)	0.07
Alcohol	0.86 (0.47, 1.58)	0.63	1.01 (0.53, 1.92)	0.97
Tobacco	0.68 (0.38, 1.21)	0.19	0.69 (0.38, 1.26)	0.23
Polysubstance score‡	0.92 (0.68, 1.26)	0.62	0.93 (0.63, 1.30)	0.66
Polysubstance Category§				
Neither	REF		REF	
Smoking, no other substances	0.50 (0.25, 1.02)	0.06	0.49 (0.23, 1.04)	0.06
No smoking, other substances	0.66 (0.34, 1.27)	0.22	0.64 (0.30, 1.36)	0.25
Both	1.14 (0.55, 2.36)	0.72	1.19 (0.56, 2.52)	0.65

* Hypertensive disorders include pre-eclampsia, eclampsia, gestational hypertension, and chronic hypertension.

† Substance use effects were estimated controlling for concurrent substance use, each model included the 4 other substances used during pregnancy.

‡ The polysubstance score was defined as the count of substances used (reference was using 0 substances during pregnancy, ranging to using all 5 substances during pregnancy), controlling for only sociodemographic covariates.

§ Polysubstance use categories were defined as: (1) using no substances during pregnancy (“neither”), (2) only smoking tobacco during pregnancy but no other substances, (3) using opioids, cocaine, or alcohol during pregnancy but no smoking, or (4) smoking and using either opioids, cocaine, or alcohol during pregnancy (“both”).

∥ Adjusted for maternal age, maternal pre-pregnancy BMI, yearly income, educational status, immigration history, parity, and sex of the child. BMI, body mass index; CI, confidence interval.

For Hispanic participants (Table 3), the only suggestive association between substance use with hypertensive disorders was for cannabis exposure during pregnancy, which increased the odds of hypertensive disorders 2.66 times (95% CI: 0.92, 7.64), after adjustment, though this did not reach statistical significance. For pre-eclampsia only, cannabis had a similar effect size (OR: 2.69, 95% CI: 0.91, 7.92), though this overlapped with the null as well. In the model examining smoking and concurrent polysubstance use, smoking without concurrent use during pregnancy had a more pronounced protective effect (OR: 0.50, 95% CI: 0.25, 1.02) though this also was not statistically significant.

When we restricted to primiparous participants in order to investigate confounding by indication in prior pregnancy, we continued to see reduced odds (OR: 0.60, 95% CI: 0.37, 0.96) of HTN only for participants who both smoked tobacco and used other substances (Table 4). We did not find any significant associations for pre-eclampsia specifically in this subsample.

**Table 4:** Substance use during pregnancy and association with hypertensive disorders and pre-eclampsia among primiparous Black and Hispanic participants.

	Hypertensive disorders*		Pre-eclampsia (mild or severe)	
Unadjusted models	Odds ratio (95% CI)	*P* value	Odds ratio (95% CI)	*P* value
Opioids	2.04 (0.96, 4.34)	0.06	1.53 (0.61, 3.79)	0.36
Cocaine	0.44 (0.14, 1.42)	0.17	0.45 (0.10, 2.01)	0.29
Cannabis	1.02 (0.59, 1.76)	0.94	1.17 (0.61, 2.24)	0.63
Alcohol	0.81 (0.56, 1.17)	0.27	1.07 (0.70, 1.62)	0.75
Tobacco	0.89 (0.67, 1.17)	0.39	0.86 (0.61, 1.20)	0.37
Polysubstance score	0.88 (0.75, 1.03)	0.12	0.91 (0.75, 1.11)	0.36
Polysubstance category†				
Neither	REF		REF	
Smoking, no other substances	0.98 (0.72, 1.33)	0.87	0.97 (0.67, 1.40)	0.87
No smoking, other substances	1.09 (0.80, 1.49)	0.59	1.41 (0.99, 2.01)	0.06
Both	0.72 (0.49, 1.07)	0.11	0.75 (0.46, 1.23)	0.26
	Hypertensive disorders*		Pre-eclampsia (mild or severe)	
Adjusted models^[30]^	Odds ratio (95% CI)	*P* value	Odds ratio (95% CI)	*P* value
Opioids	1.99 (0.85, 4.64)	0.11	1.49 (0.59, 3.78)	0.40
Cocaine	0.49 (0.14, 1.71)	0.26	0.67 (0.19, 2.37)	0.24
Cannabis	1.13 (0.62, 2.08)	0.68	1.20 (0.60, 2.38)	0.61
Alcohol	0.74 (0.49, 1.13)	0.16	1.09 (0.69, 1.72)	0.70
Tobacco	0.68 (0.48, 0.97)	0.03	0.73 (0.49, 1.08)	0.12
Polysubstance score	0.83 (0.69, 1.01)	0.07	0.87 (0.69, 1.09)	0.21
Polysubstance Category†				
Neither	REF		REF	
Smoking, no other substances	0.73 (0.49, 1.08)	0.12	0.87 (0.56, 1.34)	0.53
No smoking, other substances	1.09 (0.74, 1.59)	0.67	1.37 (0.91, 2.08)	0.14
Both	0.60 (0.37, 0.96)	0.03	0.60 (0.33, 1.08)	0.88

* Hypertensive disorders include pre-eclampsia, eclampsia, gestational hypertension, and chronic hypertension.

† Polysubstance use categories were defined as: (1) using no substances during pregnancy, (2) only smoking tobacco during pregnancy but no other substances, (3) using opioids, cocaine, or alcohol during pregnancy but no smoking, or (4) smoking and using either opioids, cocaine, or alcohol during pregnancy.

‡ Adjusted for maternal age, maternal pre-pregnancy BMI, yearly income, educational status, immigration history, parity, and sex of the child. We found evidence that collider-stratification bias may account for the smoking-hypertension paradox in pregnancy. BMI, body mass index; CI, confidence interval.

In the cause-specific Cox models (Table 5) to assess potential collider bias, tobacco smoking was highly associated with PTB among study participants without hypertensive disorders in our unadjusted and adjusted models. In the competing risk model, which assesses the effect of smoking on the cumulative incidence of pre-eclampsia among those who have and have not experienced the competing event (PTB), the effect creating a paradox remained (HR: 0.85, 95% CI: 0.73, 0.99). This was not statistically significant after adjustment, but the direction of effect remained (HR: 0.85, 95% CI: 0.71, 1.02). Tobacco (HR: 1.29, 95% CI: 1.08, 1.54) and opioids (HR: 1.39, 95% CI: 1.01, 1.90) were statistically associated with PTB in our sample without hypertensive disorders, and opioids were statistically associated with hypertensive disorders when adjusting for all other substances among term births (HR: 1.69, 95% CI: 1.06, 2.70). In our replication analysis (Supplementary Table 11, http://links.lww.com/PN9/A22), we found that tobacco use was associated with gestational hypertension when accounting for the competing risk of PTB (HR: 1.51, 95% CI: 1.06, 2.17); however, this was not significant after adjustment for available covariates, though the effect estimates remained in the same direction (HR: 1.22 adjusted *vs*. HR: 1.51 unadjusted).

**Table 5:** Assessment of competing risk of preterm birth for pre-eclampsia among all participants.

	PTB among no pre-eclampsia		Pre-eclampsia among no PTB		Competing risk regression for pre-eclampsia	
Adjusted models§	Hazards ratio (95% CI)	*P* value	Hazards ratio (95% CI)	*P* value	Hazards ratio* (95% CI)	*P* value
Opioids	1.39 (1.01, 1.90)	0.04	1.69 (1.06, 2.70)	0.03	1.03 (0.80, 1.34)	0.80
Cocaine	1.34 (0.85, 2.11)	0.20	0.47 (0.11, 1.97)	0.30	0.76 (0.39, 1.49)	0.43
Cannabis	1.01 (0.76, 1.34)	0.93	0.79 (0.49, 1.27)	0.33	1.11 (0.77, 1.61)	0.56
Alcohol	1.04 (0.85, 1.28)	0.68	0.95 (0.72, 1.27)	0.75	0.87 (0.71, 1.07)	0.19
Tobacco	1.29 (1.08, 1.54)	0.006	0.81 (0.63, 1.04)	0.10	0.85 (0.71, 1.02)	0.07
Polysubstance score†	1.18 (1.08, 1.29)	<0.001	0.93 (0.81, 1.08)	0.34	0.88 (0.80, 0.97)	0.01

* Fine-Gray subdistribution hazards model with preterm birth as the competing risk, and pre-eclampsia (mild, moderate, or severe) as the main outcome of interest. For all hazards models conducted, the gestational age was the time metric.

† Polysubstance score was an unweighted sum of the number of substances used in order to estimate the aggregate burden of substance use in pregnancy.

§ Adjusted for maternal age, maternal pre-pregnancy BMI, yearly income, educational status, immigration history, parity, and sex of the child. Gestational age was the time metric for Cox regression. BMI, body mass index; CI, confidence interval; PTB, preterm birth.

## Discussion

We examined whether the smoking-hypertension paradox during pregnancy persisted after addressing a range of potential confounding factors and competing risks for hypertensive outcomes in a large, multiethnic birth cohort. After stratifying by race/ethnicity, we observed a smoking-hypertensive disorders paradox among Black participants when including tobacco with other substances, but not smoking alone. Smoking, also, was not a clear risk or protective factor for these outcomes among Hispanic participants. We also found evidence that potential collider-stratification biases due to competing risks likely account for the smoking-hypertension paradox in our sample, potentially due to competing risks from PTB that left truncates the birth cohort.

We hypothesized that pregnant people who had used substances during their initial pregnancy and had been at elevated risk for hypertensive disorders, may then decide to reduce their substance use in subsequent pregnancies. This would result in confounding by indication, but while previous studies have found associations between hypertensive disorders and primiparity,^[37]^ studies restricted to primiparous participants have also found a reduced risk of HTN for smoking compared to non-smoking study participants.^[7]^ In our study, restricting to primiparous participants also did not seem to explain the paradox.

Additionally, an Icelandic birth registry study suggested that the paradox could also be explained by adjustment for gestational age by opening a collider stratification pathway to create bias.^[11]^ We assessed this by stratifying in our cause-specific hazards model by patients who had full-term births, which led to a null association between smoking and hypertensive disorders or pre-eclampsia (Supplementary Table 1, http://links.lww.com/PN9/A22). With gestational age as a time metric in our competing risk models, we found no evidence for an association between tobacco smoking in pregnancy and reduced risk of hypertensive disorders. This suggests collider-stratification may better explain the paradox than confounding by indication. This is supported by previous research that has focused on various selection biases, including left truncation bias^[38]^ and live birth bias.^[39,40]^ These selection biases can also be conceived as a collider-stratification case. Here, two otherwise unrelated factors both increase the likelihood of selection into the study. Because selection is required for inclusion in the study, it is not adjusted for and, therefore, it is left open to influence the relationship of interest. Therefore, our findings give credence that selection bias is the major driver of the paradox, and that using analytic methods that can account for selection due to gestational age, such as Cox proportional hazards or Fine-Gray hazards models, may surmount these biases.

Of note, maternal birth outcomes (eg, PTB) among Hispanic participants have been dubbed a paradox in prior studies, given they are more similar to those of non-Hispanic White participants, in contrast to many of their socioeconomic indicators that are more similar to those of non-Hispanic Black participants.^[41]^ Our analyses contrasting Black and Hispanic participants’ substance use highlights the importance of considering the particular nuances of race/ethnicity as a factor when examining exposure-outcome associations. In our competing risk analysis among both Black and Hispanic participants, we found different directions of effects—while each substance increased the risk for PTB, smoking was not associated with hypertensive disorders when restricted to full-term births. However, when using a subdistribution hazards model, the paradoxical association returned.

As in our study, other research has also examined whether race/ethnicity modifies the relationship between current smoking and hypertension. Liu *et al* found reduced risk of hypertension in pregnancy associated with smoking was present only among non-Hispanic White participants.^[33]^ Another study found reduced risk in White and American Indian (both non-Hispanic) participants <35 years of age, but among older participants, smoking increased the risk.^[42]^ That study, however, did not find associations between smoking and HTN among Black participants,^[42]^ while we did. This discrepancy may be due to differences in sample characteristics. For instance, our participants were specifically from an urban, low-income cohort of whom almost half of our Black participants were immigrants, and therefore may have different risk behaviors than other African-American populations. Research has also found that Caribbean-born Black people had reduced substance use rates compared to US-born.^[43]^ But past studies have also found reduced risk of hypertension among people born in Africa living in the US *vs*. US-born people of African descent.^[44]^ When we conducted sensitivity analyses for maternal place of birth in our stratified sample, it was an impactful confounder but its causal relationship was unclear. In this context, our results suggest nuanced differences between foreign- *vs*. US-born Black communities.

Few Hispanic participants used substances in our sample, which comports with previous findings during pregnancy.^[45]^ Despite a low prevalence in our study, cannabis exposure was a risk factor for HTN even after adjustment. Another Massachusetts study found that just under half of the Hispanic participants in their sample ceased smoking once they found out they were pregnant, but that those who continued using cannabis during pregnancy were more likely to report concurrent tobacco smoking.^[46]^ It is unclear if the association we observed is due to cannabis use itself only or to concurrent substance patterns not otherwise well-accounted for in our models.

Although the small number of Hispanic participants using cannabis alone made it difficult to untangle concurrent effects with tobacco during pregnancy, our results indicating more than twice the risk of pre-eclampsia among cannabis-using Hispanic participants may be due to a dual paradox: (a) the first, in this case, would be the “Hispanic paradox,” by which Hispanic participants on average have birth outcomes more comparable to that of White participants than Black participants^[41]^; (b) the second would be a “smoking/substance paradox,” as seen in our results which showed a reduced risk in the point estimates for HTN among Hispanic participants who only smoked or only used one substance during pregnancy. Through adjustment for concurrent substance exposure and stratification, the initial protective association between substances and HTN during pregnancy demonstrated a null association among the Hispanic sample. Further research should be done to better understand how these potential phenomena interact with one another in this and other populations.

This study’s strengths include its large sample size of substance-using participants that enabled assessment of confounding from concurrent use of multiple substances. Prior studies have usually have not taken into account a wide variety of substance use covariates.^[7,8,11,13,42,47]^ Concurrent use is often difficult to measure; however, our study utilized a Certificate of Confidentiality to increase the likelihood of valid responses and may have allowed participants to feel more comfortable disclosing substance use. In our baseline sample, 496 and 150 participants reported using ≥2 or ≥3 substances during pregnancy, respectively, which allowed for examination of their combined effects with smoking. Additionally, we had many Black and Hispanic participants that allowed us to conduct subgroup analyses among pregnant participants at high-risk for these outcomes, including maternal place of birth. The NVSS data lacked many of the variables in the BBC (which speaks to the strength of our cohort), therefore our replication of models was limited. But in our replication analyses, we found that our competing risk analysis continued to reduce the smoking-hypertension paradox to a null effect, giving further reliance to our results.

Our study had several limitations. After stratification, we had a limited sample size for certain polysubstance combinations and our power was constrained for analyses on cannabis, opioids, and cocaine. We were also limited to self-reported substance use, which may be underreported due to the stigmatized nature of use during pregnancy. However, our tobacco,^[48]^ alcohol,^[49]^ and cannabis use prevalence rates were similar to previous national estimates, including the National Survey on Drug Use and Health (NSDUH) estimates from 2015 through 2018.^[50]^ For opioids, we incorporated ICD-9^[20]^ and ICD-10^[21](p10)^ codes for NAS and NOWS into our exposure definition, resulting in a higher prevalence than the NSDUH. Ideally, use of toxicological data could further enhance the accuracy of the substance use variable. Additionally, we lacked power to examine the timing of smoking/substance exposures during specific trimesters and associations with hypertensive disorders, and though we conducted several sensitivity analyses, it is possible that other unmeasured confounders could impact our associations. Notably, we used retrospective recall in order to identify and determine substance use. Ascertainment bias towards the null due to social desirability may occur, and modeling in the BBC has suggested that underreporting may range from 15.7% to 20.2%.^[51]^ This would result in our estimates being more conservative. Additionally, we are unable to directly assess left truncation bias in our sample, given it is not possible to identify the counterfactual states of people whose pregnancies ended early but who would have gone on to experience hypertensive disorders had their pregnancy continued further into gestation. Our eligibility criteria for inclusion following a live birth further reduced our ability to investigate live birth bias, a form of left truncation bias.^[52]^ Finally, our study sample recruited from a large safety net hospital which primarily serves patients who are lower income and may be at increased risk of substance use. This is somewhat supported by the high rate of substance use within our sample compared to estimated use in the general pregnant population.^[50]^ However, this smoking-hypertension paradox has been described in a number of pregnant populations before with varying demographics, and therefore we expect our findings are generalizable to other multiethnic populations.

## Conclusions

This study examined five potential explanations for the smoking-hypertension paradox during pregnancy in a birth cohort at increased risk for both the exposure and outcome of interest. This work yields important insights into applicable epidemiological principles using a high-risk cohort not commonly represented in literature. We examined concurrent substance use as a confounder and showed a lack of effect for participants who only smoked during pregnancy. Notably, stratifying for PTB in the cohort reduced the effect representing the paradox to the null. We also examined effect modification based on race/ethnicity and found a continued paradox among Black participants but not Hispanic participants who smoked during pregnancy. This work offers new insight into the long-standing but unexplained maternal smoking-pre-eclampsia paradox and highlights the need for future studies to consider the importance of the factors we have identified as well as to pay attention to these factors in clinical practice.

## Acknowledgments

We thank the mothers, children, and families who participate in the Boston Birth Cohort for volunteering their time and responses over the years. We also thank Linda Rosen, of the Boston Medical Center Clinical Data Warehouse, whose timely data extraction from electronic medical records made this research also possible.

## Author contributions

PJS, XW, and HGD conceptualized the overall research question and study aims. XH and GW oversaw data management and cleaning. HGD analyzed the data and CLA, ZMS, and IB contributed content expertise to the interpretation of the data. HGD drafted the initial manuscript, and all authors provided substantial edits and gave final approval of the submitted version.

## Funding

The Boston Birth Cohort is supported by the National Institutes of Health grants: R01HD086013, 2R01HD041702, R01HD098232, R01ES031272, R01ES031521, and U01 ES034983. This study is supported in part by the Health Resources and Services Administration of the U.S. Department of Health and Human Services, grant number UT745949, Autism Single Investigator Innovation Program. The funding agencies had no involvement in the collection, analysis, or interpretation of data; in the writing of the report; or in the decision to submit the article for publication.

## Conflicts of Interest

The authors declare that they have no competing interests.

## Supplementary Material


