# Supplementary material for: Addressing the smoking-hypertension paradox in pregnancy: insight from a multiethnic US birth cohort
Source: Precis Nutr. 2023 May 24;2(2):e00035. doi: 10.1097/PN9.0000000000000035 (PMC10312115; doi:10.1097/PN9.0000000000000035)
Supplement: Supplementary file 1 [file pn9-2-e00035-s001.pdf]

Supplementary Material.

Supplemental Table 1. Substance use during pregnancy and association with hypertensive disorders and pre-eclampsia among White participants. (N= 1007)

	Hypertensive Disorders[1]		Pre-Eclampsia (Mild or Severe)	
Unadjusted	Odds Ratio (95% CI)	P-value	Odds Ratio (95% CI)	P-value
Opioids[2]	1.13 (0.71, 1.79)	0.60	0.89 (0.50, 1.59)	0.70
Cocaine	0.71 (0.19, 2.67)	0.61	[Failed to converge]	
Cannabis	0.45 (0.13, 1.53)	0.20	[Failed to converge]	
Alcohol	0.77 (0.44, 1.37)	0.38	1.08 (0.54, 2.17)	0.82
Tobacco	0.77 (0.51, 1.17)	0.22	0.49 (0.28, 0.85)	0.01
Polysubstance score[3]	0.89 (0.72, 1.11)	0.31	0.74 (0.56, 0.99)	0.04
Polysubstance Category[4]				
Neither	REF		REF	
Smoking, no other substances	0.49 (0.28, 0.85)	0.01	0.61 (0.30, 1.21)	0.16
No smoking, other substances	0.62 (0.31, 1.24)	0.17	1.10 (0.51, 2.39)	0.80
Both	0.71 (0.44, 1.15)	0.16	0.49 (0.25, 0.95)	0.04
	Hypertensive Disorders[1]		Pre-Eclampsia (Mild or Severe)	
Adjusted[5]	Odds Ratio (95% CI)	P-value	Odds Ratio (95% CI)	P-value
Opioids	1.34 (0.79, 2.25)	0.27	1.27 (0.65, 2.46)	0.48
Cocaine	1.03 (0.26, 4.02)	0.97	[Failed to converge]	
Cannabis	0.41 (0.11, 1.47)	0.17	[Failed to converge]	
Alcohol	0.70 (0.39, 1.28)	0.25	1.06 (0.52, 2.15)	0.87
Tobacco	0.93 (0.58, 1.49)	0.76	0.60 (0.32, 1.11)	0.11
Polysubstance score[3]	0.94 (0.74, 1.20)	0.64	0.84 (0.61, 1.14)	0.26
Polysubstance Category[4]				
Neither	REF		REF	

Smoking, no other substances	0.51 (0.27, 0.96)	0.04	0.65 (0.31, 1.39)	0.27
No smoking, other substances	0.54 (0.26, 1.14)	0.11	1.12 (0.51, 2.49) 27	0.78
Both	0.73 (0.42, 1.26)	0.25	0.59 (0.27, 2.49)	0.17

[1]Hypertensive disorders include pre-eclampsia, eclampsia, gestational hypertension, and chronic hypertension

[2]Substance use effects were estimated controlling for concurrent substance use, each model included the 4 other substances used during pregnancy.

[3]The polysubstance score was defined as the count of substances used (reference was using 0 substances during pregnancy, ranging to using all 5 substances during pregnancy), controlling for only sociodemographic confounders.

[4]Polysubstance use categories were defined as: (1) using no substances during pregnancy ("neither"), (2) only smoking tobacco during pregnancy but no other substances, (3) using opioids, cocaine, or alcohol during pregnancy but no smoking, or (4) smoking and using either opioids, cocaine, or alcohol during pregnancy ("both").

[5]Adjusted for maternal age, maternal pre-pregnancy BMI, yearly income, educational status, immigration history, parity, and sex of the child.

Supplementary Table 2. Polysubstance categories of smoking and other substance use and odds of hypertensive disorders in pregnancy.

Smoking	Opioids	Odds Ratio (95% CI)	P-value	Odds Ratio (95% CI)	P-value	Odds Ratio (95% CI)	P-value
No	No	REF		REF		REF	
Yes	No	0.70 (0.52, 0.94)	0.02	0.62 (0.42, 0.92)	0.02	0.89 (0.59, 1.36)	0.59
No	Yes	4.92 (1.71, 14.16)	0.003	1.91 (0.54, 6.81)	0.32	8.21 (2.90, 23.24)	<0.001
Yes	Yes	1.49 (0.53, 4.1)	0.45	0.90 (0.21, 3.91)	0.89	3.28 (1.08, 9.96)	0.04
Smoking	Cannabis	Odds Ratio (95% CI)	P-value	Odds Ratio (95% CI)	P-value	Odds Ratio (95% CI)	P-value
No	No	REF		REF		REF	
Yes	No	0.66 (0.47, 0.94)	0.02	0.58 (0.37, 0.91)	0.02	1.02 (0.66, 1.57)	0.94
No	Yes	0.91 (0.54, 1.51)	0.71	1.01 (0.56, 1.82)	0.97	0.68 (0.30, 1.57)	0.37
Yes	Yes	0.91 (0.52, 1.59)	0.74	0.80 (0.40, 1.60)	0.52	0.78 (0.34, 1.80)	0.56
Smoking	Alcohol	Odds Ratio (95% CI)	P-value	Odds Ratio (95% CI)	P-value	Odds Ratio (95% CI)	P-value
No	No	REF		REF		REF	
Yes	No	0.69 (0.48, 0.98)	0.04	0.51 (0.32, 0.83)	0.006	0.89 (0.56, 1.41)	0.62
No	Yes	1.05 (0.75, 1.48)	0.77	1.32 (0.91, 1.92)	0.15	1.07 (0.66, 1.73)	0.79
Yes	Yes	0.86 (0.49, 1.51)	0.59	1.09 (0.58, 2.04)	0.80	1.25 (0.62, 2.52)	0.53

Supplementary Table 3. Logistic regression sensitivity analysis of spontaneous preterm birth, pre-eclampsia, and the composite outcome.

	Spontaneous PTB[1]		Pre-eclampsia among no PTB		Composite Outcome	
Unadjusted	Odds Ratio (95% CI)	P-value	Odds Ratio (95% CI)	P-value	Odds Ratio (95% CI)	P-value
Opioids	1.83 (1.42, 2.36)	<0.001	0.88 (0.46, 1.68)	0.70	1.43 (1.15, 1.79)	0.001
Cocaine	1.90 (1.18, 3.06)	0.008	0.33 (0.05, 2.37)	0.27	1.20 (0.78, 1.85)	0.41
Cannabis	1.68 (1.30, 2.18)	<0.001	0.86 (0.45, 1.64)	0.65	1.26 (1.00, 1.58)	0.05
Alcohol	1.25 (1.03, 1.51)	0.02	1.47 (1.03, 2.09)	0.03	1.14 (0.97, 1.34)	0.10
Tobacco	1.75 (1.54, 2.00)	<0.001	0.84 (0.62, 1.14)	0.26	1.30 (1.16, 1.45)	<0.001
Polysubstance score[4]	1.34 (1.25, 1.45)	<0.001	0.97 (0.82, 1.16)	0.78	1.15 (1.08, 1.23)	<0.001
	Spontaneous PTB		Pre-eclampsia among no PTB		Composite Outcome	
Adjusted[3]	Odds Ratio (95% CI)	P-value	Odds Ratio (95% CI)	P-value	Odds Ratio (95% CI)	P-value
Opioids	1.41 (1.06, 1.89)	0.02	1.04 (0.51, 2.12)	0.92	1.32 (1.03, 1.70)	0.03
Cocaine	1.29 (0.79, 2.10)	0.30	0.35 (0.05, 2.59)	0.31	0.98 (0.62, 1.52)	0.91
Cannabis	1.32 (1.01, 1.73)	0.04	0.84 (0.43, 1.62)	0.60	1.07 (0.85, 1.36)	0.56
Alcohol	1.08 (0.89, 1.31)	0.45	1.48 (1.03, 2.14)	0.35	1.03 (0.88, 1.22)	0.69
Tobacco	1.43 (1.23, 1.67)	<0.001	0.76 (0.54, 1.07)	0.12	1.11 (0.97, 1.27)	0.12
Polysubstance score	1.20 (1.10, 1.31)	<0.001	0.98 (0.80, 1.20)	0.84	1.07 (0.99, 1.15)	0.10

[1]Preterm birth (PTB).

[2]Composite outcome included either having spontaneous PTB or pre-eclampsia (including mild or severe).

[3]Adjusted for maternal age, maternal pre-pregnancy BMI, yearly income, educational status, immigration history, parity, and sex of the child.

[4]Polysubstance score was the number of substances used during pregnancy, and may be interpreted as the additive effect of using an additional substance during pregnancy.

Supplementary Table 4. Adjusted associations between substance use and stressful life events with hypertensive disorders, pre-eclampsia, and chronic hypertension among Black participants.

Adjusted		Hypertensive Disorders		Pre-Eclampsia (Mild or Severe)		Chronic HTN	
		Odds Ratio (95% CI)	P-value	Odds Ratio (95% CI)	P-value	Odds Ratio (95% CI)	P-value
Substance use	Opioids	2.58 (1.22, 5.45)	0.01	1.34 (0.51, 3.53)	0.55	5.21 (2.28, 11.86)	<0.001
	Cocaine	0.70 (0.23, 2.09)	0.52	0.43 (1.02, 1.84)	0.26	0.23 (0.03, 1.77)	0.16
	Cannabis	0.94 (0.62, 1.44)	0.78	0.90 (0.56, 1.45)	0.68	0.89 (0.47, 1.68)	0.72
	Alcohol	0.93 (0.68, 1.28)	0.66	1.21 (0.86, 1.70)	0.28	1.05 (0.67, 1.65)	0.81
	Tobacco	0.67 (0.49, 0.93)	0.02	0.55 (0.37, 0.83)	0.004	0.89 (0.57, 1.39)	0.61
	Polysubstance score	0.91 (0.77, 1.07)	0.24	0.90 (0.75, 1.08)	0.25	1.02 (0.82, 1.27)	0.84
Combined smoking and other substance							
Smoking	Opioids						
No	No	REF		REF		REF	
Yes	No	0.65 (0.46, 0.89)	0.008	0.54 (0.35, 0.81)	0.003	0.80 (0.50, 1.27)	0.34
No	Yes	3.24 (1.09, 9.66)	0.03	1.49 (0.41, 5.40)	0.54	5.54 (1.80, 17.06)	0.003
Yes	Yes	1.85 (0.63, 5.40)	0.26	1.00 (0.23, 4.41)	1.00	4.55 (1.36, 15.20)	0.02
Smoking	Cannabis						
No	No	REF		REF		REF	
Yes	No	0.60 (0.41, 0.87)	0.007	0.49 (0.30, 0.80)	0.004	0.89 (0.54, 1.45)	0.63
No	Yes	0.84 (0.49, 1.46)	0.55	0.90 (0.49, 1.67)	0.75	0.89 (0.36, 1.45)	0.76
Yes	Yes	0.92 (0.41, 1.65)	0.77	0.74 (0.36, 1.51)	0.41	0.88 (0.36, 2.11)	0.77
Smoking	Alcohol						
No	No	REF		REF		REF	
Yes	No	0.64 (0.43, 0.93)	0.02	0.46 (0.28, 0.76)	0.002	0.83 (0.50, 1.39)	0.48
No	Yes	0.92 (0.64, 1.32)	0.65	1.20 (0.81, 1.76)	0.36	1.01 (0.60, 1.70)	0.98
Yes	Yes	7.75 (0.42, 1.42)	0.41	0.91 (0.47, 1.75)	0.36	1.08 (0.50, 2.33)	0.85
Stressful life events	General stress	1.20 (1.05, 1.37)	0.006	1.34 (1.14, 1.57)	<0.001	1.31 (1.08, 1.59)	0.006
	Index	1.15 (1.02, 1.29)	0.02	1.24 (1.08, 1.43)	0.002	1.23 (1.04, 1.47)	0.02

Stress score	1.03 (1.00, 1.06)	0.04	1.05 (1.02, 1.09)	0.003	1.01 (0.98, 1.06)	0.35

Adjusted for parity, maternal place of birth, maternal age, maternal education level, maternal income quartile, maternal pre-pregnancy BMI, child sex and child birth year (for temporal trends).

Note: The reference for substance use is simply not using the substance. Women may be using other substances in the reference group though.

Supplementary Table 5. Adjusted associations between substance use and stressful life events with hypertensive disorders, pre-eclampsia, and chronic hypertension among Hispanic participants.

Adjusted		Hypertensive Disorders		Pre-Eclampsia (Mild or Severe)		Chronic HTN	
		Odds Ratio (95% CI)	P-value	Odds Ratio (95% CI)	P-value	Odds Ratio (95% CI)	P-value
Substance use	Opioids	0.65 (0.17, 2.44)	0.52	0.46 (0.11, 2.00)	0.30	1.14 (0.24, 5.46)	0.87
	Cocaine	0.68 (0.10, 4.45)	0.69	0.60 (0.08, 4.72)	0.63	1.74 (0.20, 1.51)	0.61
	Cannabis	2.41 (1.00, 5.84)	0.05	2.32 (0.93, 5.82)	0.07	15.78 (5.45, 45.66)	<0.001
	Alcohol	0.74 (0.48, 1.46)	0.53	0.91 (0.50, 1.66)	0.76	1.12 (0.43, 2.94)	0.82
	Tobacco	0.91 (0.47, 1.77)	0.78	0.78 (0.38, 1.58)	0.49	2.57 (1.07, 6.15)	0.03
	Polysubstance score	0.97 (0.71, 1.32)	0.84	0.94 (0.68, 1.28)	0.69	1.60 (1.10, 2.32)	0.01
Combined smoking and other substance							
Smoking	Opioids						
No	No	REF		REF		REF	
Yes	No	0.95 (0.47, 1.95)	0.90	0.87 (0.40, 1.88)	0.72	2.92 (1.15, 7.40)	0.02
No	Yes	[X]		[X]		[X]	
Yes	Yes	0.75 (0.19, 2.91)	0.67	0.41 (0.12, 2.27)	0.38	1.66 (0.33, 8.25)	0.54
Smoking	Cannabis						
No	No	REF		REF		REF	
Yes	No	0.58 (0.25, 1.37)	0.21	0.44 (0.17, 1.14)	0.09	0.85 (0.24, 3.06)	0.80
No	Yes	0.74 (0.09, 5.80)	0.77	0.79 (0.10, 6.25)	0.82	[X]	
Yes	Yes	3.61 (1.33, 9.75)	0.01	3.19 (1.13, 9.01)	0.03	27.51 (8.68, 87.20)	<0.001
Smoking	Alcohol						
No	No	REF		REF		REF	
Yes	No	0.75 (0.34, 1.63)	0.46	0.55 (0.23, 1.32)	0.18	2.87 (1.15, 7.17)	0.02
No	Yes	0.70 (0.38, 1.28)	0.25	0.71 (0.35, 1.45)	0.35	1.21 (0.41, 3.56)	0.73
Yes	Yes	1.68 (0.53, 5.32)	0.37	1.94 (0.63, 6.02)	0.25	1.50 (0.18, 12.59)	0.71
Stressful life events	General stress	1.01 (0.82, 1.25)	0.90	1.16 (0.94, 1.46)	0.20	1.30 (0.88, 1.89)	0.18
	Index	1.09 (0.90, 1.32)	0.37	1.20 (0.98, 1.47)	0.08	1.55 (1.11, 2.18)	0.01

Stress score	1.01 (0.98, 1.05)	0.51	0.99 (0.95, 1.04)	0.69	1.08 (1.00, 1.16)	0.05

Adjusted for parity, maternal place of birth, maternal age, maternal education level, maternal income quartile, maternal pre-pregnancy BMI, child sex and child birth year (for temporal trends).

Note: The reference for substance use is simply not using the substance. Women may be using other substances in the reference group though.

Supplementary Table 6. Sequential adjustment for covariates and the impact on the estimated effect of smoking during pregnancy and pre-eclampsia.

Sex of Child	Maternal Education	Maternal Marital Status	Maternal Place of Birth	Parity	Yearly Income	Body Mass Index	Maternal Age	Smoking estimate[1]
								0.93 (0.74, 1.16)[2]
X								0.89 (0.70, 1.11)
	X							0.89 (0.71, 1.13)
		X						0.89 (0.70, 1.12)
			X					0.77 (0.60, 1.00)
				X				0.90 (0.72, 1.14)
					X			0.88 (0.70, 1.11)
						X		0.84 (0.67, 1.06)
							X	0.93 (0.74, 1.17)

[1]Competing risk regression estimate using Fine-Gray subdistribution model to adjust for spontaneous preterm birth, using gestational age as the time metric.

[2]Unadjusted estimate.

Supplementary Table 7. Assessment of competing risk of preterm birth for hypertensive disorders among all participants.

	PTB[1] among no Hypertensive Disorders		Hypertensive Disorders among no PTB		Competing Risk Regression[2] for Hypertensive Disorders	
Unadjusted	Hazards Ratio (95% CI)	P-value	Hazards Ratio (95% CI)	P-value	Hazards Ratio (95% CI)	P-value
Opioids	2.67 (2.19, 3.25)	<0.001	1.76 (1.17, 2.65)	0.008	1.23 (0.87, 1.73)	0.25
Cocaine	1.33 (0.93, 1.92)	0.12	0.75 (0.28, 1.98)	0.56	0.94 (0.49, 1.80)	0.84
Cannabis	1.74 (1.41, 2.15)	<0.001	1.25 (0.84, 1.88)	0.28	1.05 (0.73, 1.51)	0.79
Alcohol	1.34 (1.14, 1.56)	<0.001	1.05 (0.81, 1.36)	0.70	0.97 (0.76, 1.25)	0.83
Tobacco	1.45 (1.28, 1.65)	<0.001	0.83 (0.65, 1.06)	0.14	0.73 (0.57, 0.92)	0.007
Polysubstance score[3]	1.30 (1.22, 1.37)	<0.001	1.02 (0.90, 1.15)	0.76	0.94 (0.84, 1.06)	0.31
	PTB[1] among no Hypertensive Disorders		Hypertensive Disorders among no PTB		Competing Risk Regression[2] for Hypertensive Disorders	
Adjusted[4]	Hazards Ratio (95% CI)	P-value	Hazards Ratio (95% CI)	P-value	Hazards Ratio (95% CI)	P-value
Opioids	2.24 (1.82, 2.75)	<0.001	1.95 (1.28, 2.96)	0.002	1.40 (0.98, 2.01)	0.07
Cocaine	1.00 (0.69, 1.45)	1.00	0.77 (0.29, 2.08)	0.61	1.08 (0.55, 2.12)	0.82
Cannabis	1.65 (1.33, 2.04)	<0.001	1.39 (0.91, 2.12)	0.12	1.22 (0.85, 1.76)	0.29
Alcohol	1.22 (1.04, 1.43)	0.02	0.96 (0.74, 1.26)	0.77	0.97 (0.76, 1.25)	0.84
Tobacco	1.30 (1.13, 1.49)	<0.001	0.81 (0.62, 1.05)	0.14	0.77 (0.61, 0.99)	0.04
Polysubstance score	1.24 (1.16, 1.32)	<0.001	1.01 (0.88, 1.16)	0.84	0.99 (0.87, 1.12)	0.84

[1]PTB: Preterm Birth

[2]Fine-Gray subdistribution hazards model with spontaneous preterm birth as the competing risk, and pre-eclampsia (mild, moderate, or severe) as the main outcome of interest.

[3]Polysubstance score was an unweighted sum of the number of substances used in order to estimate the aggregate burden of substance use in pregnancy.

[4]Adjusted for maternal age, maternal pre-pregnancy BMI, yearly income, educational status, immigration history, parity, and sex of the child.

Supplemental Table 8. E-Values for statistically significant adjusted odds ratio findings among Black participants, and among White participants.

E-Values of Significant Adjusted Model Estimates	Black participants	White participants
	Point Estimate (Lower/Upper limit)	Point Estimate (Lower/Upper limit)
Opioids[2]	5.93 (2.01)	Not sig
Cocaine	Not sig	Not sig
Cannabis	Not sig	Not sig
Alcohol	Not sig	Not sig
Tobacco	Not sig	2.30 (1.21)
Polysubstance score[3]	1.67 (1.00)	Not sig
Polysubstance Category[4]		
Neither	REF	REF
Smoking, no other substances	Not sig	Not sig
No smoking, other substances	Not sig	Not sig
Both	2.66 (1.36)	2.72 (1.25)

Note: E-Values were only generated for statistically significant estimates of effect among models adjusted for maternal age, maternal pre-pregnancy BMI, yearly income, educational status, immigration history, parity, and sex of the child. Estimates among the Hispanic participant subcohort were not statistically significant and therefore not included in this analysis.

Supplemental Table 9. E-Values for statistically significant adjusted hazards ratios of competing risk of preterm birth for pre-eclampsia among all participants.

E-Values of Significant Adjusted Model Estimates	PTB[1] among no Pre-Eclampsia	Pre-eclampsia among no PTB	Competing Risk Regression[2] for pre-eclampsia
	Point Estimate (Lower/Upper limit)	Point Estimate (Lower/Upper limit)	Point Estimate (Lower/Upper limit)
Opioids	2.13 (1.11)	2.77 (1.31)	Not sig
Cocaine	Not sig	Not sig	Not sig
Cannabis	Not sig	Not sig	Not sig
Alcohol	Not sig	Not sig	Not sig
Tobacco	1.90 (1.37)	Not sig	Not sig
Polysubstance score	1.64 (1.37)	Not sig	1.53 (1.21)

Note: E-Values were only generated for statistically significant estimates of effect among models adjusted for maternal age, maternal pre-pregnancy BMI, yearly income, educational status, immigration history, parity, and sex of the child.

Unadjusted	PTB[1] among no Hypertensive Disorders		Hypertensive Disorders among no PTB		Competing Risk Regression[2] for Hypertensive Disorders	
	Hazards Ratio (95% CI)	P-value	Hazards Ratio (95% CI)	P-value	Hazards Ratio (95% CI)	P-value
Opioids	2.67 (2.19, 3.25)	<0.001	1.76 (1.17, 2.65)	0.008	1.23 (0.87, 1.73)	0.25
Cocaine	1.33 (0.93, 1.92)	0.12	0.75 (0.28, 1.98)	0.56	0.94 (0.49, 1.80)	0.84
Cannabis	1.74 (1.41, 2.15)	<0.001	1.25 (0.84, 1.88)	0.28	1.05 (0.73, 1.51)	0.79
Alcohol	1.34 (1.14, 1.56)	<0.001	1.05 (0.81, 1.36)	0.70	0.97 (0.76, 1.25)	0.83
Tobacco	1.45 (1.28, 1.65)	<0.001	0.83 (0.65, 1.06)	0.14	0.73 (0.57, 0.92)	0.007
Polysubstance score[3]	1.30 (1.22, 1.37)	<0.001	1.02 (0.90, 1.15)	0.76	0.94 (0.84, 1.06)	0.31

Adjusted[4]	PTB[1] among no Hypertensive Disorders		Hypertensive Disorders among no PTB		Competing Risk Regression[2] for Hypertensive Disorders	
	Hazards Ratio (95% CI)	P-value	Hazards Ratio (95% CI)	P-value	Hazards Ratio (95% CI)	P-value
Opioids	2.24 (1.82, 2.75)	<0.001	1.95 (1.28, 2.96)	0.002	1.40 (0.98, 2.01)	0.07
Cocaine	1.00 (0.69, 1.45)	1.00	0.77 (0.29, 2.08)	0.61	1.08 (0.55, 2.12)	0.82
Cannabis	1.65 (1.33, 2.04)	<0.001	1.39 (0.91, 2.12)	0.12	1.22 (0.85, 1.76)	0.29
Alcohol	1.22 (1.04, 1.43)	0.02	0.96 (0.74, 1.26)	0.77	0.97 (0.76, 1.25)	0.84
Tobacco	1.30 (1.13, 1.49)	<0.001	0.81 (0.62, 1.05)	0.14	0.77 (0.61, 0.99)	0.04
Polysubstance score	1.24 (1.16, 1.32)	<0.001	1.01 (0.88, 1.16)	0.84	0.99 (0.87, 1.12)	0.84
